# Dynamic cerebral autoregulation quantification with spontaneous arterial blood pressure oscillations: Is transfer function analysis our best option?

**DOI:** 10.1113/EP091900

**Published:** 2024-04-14

**Authors:** Patrice Brassard, Jonathan D. Smirl

**Affiliations:** ^1^ Department of Kinesiology, Faculty of Medicine Université Laval Québec Canada; ^2^ Research Center of the Institut universitaire de cardiologie et de pneumologie de Québec Québec Canada; ^3^ Sport Injury Prevention Research Centre, Faculty of Kinesiology University of Calgary Calgary Alberta Canada; ^4^ Cerebrovascular Concussion Laboratory, Faculty of Kinesiology University of Calgary Calgary Alberta Canada; ^5^ Hotchkiss Brain Institute University of Calgary Calgary Alberta Canada

**Keywords:** arterial blood pressure, cerebral blood velocity, dynamic cerebral autoregulation, reliability, spontaneous oscillations, transfer function analysis

1

Transfer function analysis (TFA) is a widely used analytical method to quantify dynamic cerebral autoregulation (dCA), which represents the ability of the cerebrovasculature to buffer transient changes in arterial blood pressure (ABP). This analytical approach estimates metrics reflecting the dynamic behaviour of dCA, assuming the latter can be represented as a linear control system. The variables computed from TFA are coherence {i.e., fraction of input signal [e.g., ABP] linearly related to output signal [e.g., cerebral blood velocity (CBv)]}, gain (i.e., CBv amplitude change for a given ABP change) and phase (i.e., difference in timing of ABP and CBv waveforms). Quantification of TFA for dCA can be completed using large transient‐driven and spontaneous ABP oscillations (Claassen et al., 2016; Panerai et al., 2023). The use of spontaneous ABP fluctuations is appealing to physiologists and clinicians to assess dCA in diverse clinical contexts, where forced oscillations in ABP are not feasible or appear unsafe.

Although acknowledged, it is now clearly documented that spontaneous TFA has a poor signal‐to‐noise ratio and greatly reduced reproducibility in comparison to forced oscillations (e.g., induced by repeated squat–stand manoeuvres). This considerably limits interpretation with respect to the linear association between ABP and CBv for spontaneous TFA measures (Smirl et al., 2015). Additionally, dCA is not necessarily constant over time (i.e., a non‐stationary phenomenon), which can further affect the reproducibility of spontaneous ABP and CBv fluctuations as a result of measurement errors or the inherent physiological variability resulting from ABP changes (e.g., carbon dioxide, autonomic nervous activity, neuronal activation, body temperature, intracranial pressure, intrathoracic pressure and blood rheology), which can partly explain this phenomenon (Panerai, 2014).

The findings reported in this issue of *Experimental Physiology* by Olsen et al. (2024) add to the existing literature supporting the poor reliability of spontaneous TFA metrics. In their analysis, these authors examined the test–retest reliability of TFA metrics quantified using spontaneous ABP and CBv oscillations and based on 5 min recordings in healthy male volunteers, patients with subarachnoid haemorrhage and patients with sepsis. Absolute (using Bland–Altman analysis‐based limits of agreement and the closely related smallest real difference) and relative (using the coefficient of variation and the intraclass correlation coefficient) reliability were evaluated by comparing: (1) consecutive 5 min recordings; and (2) the first and the last 5 min of each recording. The authors reported that temporal separation of 5 min recordings of ABP and CBv spontaneous oscillations decreased absolute and relative reliability estimates of key TFA metrics (gain and normalized gain in patients with subarachnoid haemorrhage and phase for patients with sepsis), in compared to healthy male participants. The results presented by Olsen et al. (2024) have important implications for within‐day studies with multiple evaluations. Also, given that spontaneous TFA metrics have poor reliability when measurements are only a few minutes apart, one can easily imagine these problematic issues being augmented for protocols with visits on multiple days!

One strategy to overcome issues such as non‐stationarity and low signal‐to‐noise ratio between ABP and CBv is to prolong the recording duration of spontaneous oscillations. The Cerebrovascular Research Network (CARNet) White Papers mention that recordings of spontaneous oscillations in ABP and CBv should last ≥5 min, assuming stationary physiological conditions and uninterrupted good‐quality data, to improve confidence in robust TFA metric estimates and enhance frequency resolution (Claassen et al., 2016; Panerai et al., 2023). In their analysis, Olsen et al. (2024) identified their minimal recommended recording length using the point of stabilization (with the expanding window sensitivity method obtained on a 15 min recording), in addition to comparing the first half and last half of all recordings with a duration at least twice the CARNet‐recommended recording length. They reported that 5 min of continuous recording of spontaneous oscillations in ABP and CBv seems to be insufficient for stable and reliable TFA metrics, specifically in patients with subarachnoid haemorrhage and sepsis. Also, the authors found that prolonging the duration of consecutive recordings did not impact TFA metric reliability in all groups. Accordingly, although extending the recording period beyond the CARNet‐recommended 5 min did not dramatically affect the reliability of spontaneous TFA metrics, they suggested that a 5 min recording period might not be the optimal duration in the groups studied.

We congratulate the authors for this within‐day reliability analysis, which reinforces key issues present in the dCA literature associated with spontaneous oscillation TFA metrics. As recommended in the updated CARNet White Paper (Panerai et al., 2023), if one wants to overcome the influence of non‐linearity, non‐stationarity and/or the low signal‐to‐noise ratio between spontaneous ABP and CBv oscillations (and, ultimately, be more confident in using TFA), it is best to create augmented ABP fluctuations using diverse methods, such as repeated squat–stands (or sit–stands), whenever possible. This will improve the linearity between ABP and CBv and TFA interpretability. Although we agree that forcing ABP and CBv using repeated squat–stands (or sit–stands) might not always be feasible owing to mobility impairment, disabilities or for participant safety, passive methods to force ABP oscillations exist (e.g., oscillatory lower‐body negative pressure) and could be used in those situations.

Nonetheless, the findings by Olsen et al. (2024) need to be interpreted in light of important limitations. Carbon dioxide is a known and potent regulator of CBv and is widely known to influence dCA; this variable needs to be measured continuously in all dCA studies. Unfortunately, carbon dioxide levels were not monitored in the different studies included in this retrospective analysis, which impacts interpretability. Additional studies will be necessary to examine the influence of this key variable, which most probably contributes to non‐stationarity and greatly affects the reliability of spontaneous TFA metrics, especially in clinical populations. In addition, although the medications taken by patients with subarachnoid haemorrhage and sepsis were not changed throughout the recording periods, findings reported by Olsen et al. (2024) might nonetheless be affected by medications administered to these patients, especially those influencing cerebrovascular tone at baseline (i.e., at the beginning of the recording).

In summary, this retrospective analysis examined the reliability of spontaneous TFA metrics in healthy male volunteers and patients with subarachnoid haemorrhage and sepsis. These findings support existing evidence highlighting the poor within‐day reliability of TFA metrics using spontaneous ABP and CBv. Although extending the recording period beyond 5 min did not affect the reliability of spontaneous TFA metrics, it was noted that the CARNet‐recommended recording period might not represent the optimal monitoring period. In light of these findings, if researchers or clinicians cannot force ABP and CBv oscillations to use TFA for dCA quantification confidently, we suggest exploring other analytical strategies for spontaneous ABP and CBv, such as wavelet synchronization, which accounts for non‐stationarity.

## AUTHOR CONTRIBUTIONS

Both authors have approved the final version of the manuscript and agree to be accountable for all aspects of the work in ensuring that questions related to the accuracy or integrity of any part of the work are appropriately investigated and resolved. All persons designated as authors qualify for authorship, and all thosewho qualify for authorship are listed.

## CONFLICT OF INTEREST

None.

## FUNDING INFORMATION

None.
